# Food Consumption and Food Security during the COVID‐19 Pandemic in Addis Ababa

**DOI:** 10.1111/ajae.12206

**Published:** 2021-02-23

**Authors:** Kalle Hirvonen, Alan de Brauw, Gashaw T. Abate

**Keywords:** COVID‐19, food consumption, food security, nutrition security, D12, O12, Q18

## Abstract

International humanitarian organizations have expressed substantial concern about the potential for increases in food insecurity resulting from the COVID‐19 pandemic. We use a unique panel survey of a representative sample households in Addis Ababa to study both food security and food consumption during the pandemic. In contrast to some other countries in the region, Ethiopia never went into a full lockdown severely restricting movement. Despite subjective income measures suggesting a large proportion of households have been exposed to job loss or reduced incomes, we find that relative to a survey conducted in August and September of 2019, food consumption and household dietary diversity are largely unchanged or slightly increased by August 2020. We find some changes in the composition of food consumption, but they are not related to shocks found in previous phone surveys conducted with the same households. The results therefore suggest the types of subjective questions about income typically being asked in COVID‐19 phone surveys may not appropriately reflect the magnitude of such shocks. They also imply, at least indirectly, that in the aggregate food value chains have been resilient to the shock associated with the pandemic.

The COVID‐19 pandemic has led to substantial concern about threats to food security (Laborde et al. 2020a). Food prices rose almost immediately (Torero 2020), and as a result there has been substantial concern that poverty and food insecurity will rise, and the nutritional status of vulnerable groups will fall, as the pandemic continues (Laborde, Martin, and Vos 2020b). In April 2020, the World Food Programme projected the number of acutely food insecure people in the world could double by the end of 2020 without concerted action (World Food Programme 2020).

There are several ways the COVID‐19 pandemic may increase food insecurity in low and middle‐income countries. Restrictions on movement may have had the largest early negative impact on food security (Béné 2020; Resnick 2020). Devereux, Béné, and Hoddinott (2020) suggest disruptions to food systems from the pandemic both related to the food production side (production and processing) and demand side (economic and physical access to food) could negatively affect food security. Informal markets may be more disrupted than formal markets, and of particular concern on the demand side is the way that value chains function within countries (Reardon, Bellemare, and Zilberman 2020). If value chains are disrupted, then prices for disrupted chains are likely to rise; however, if value chains quickly find ways to be resilient to the pandemic, then the shock of movement restrictions that bound most heavily early in the pandemic may not have longer term effects on prices (Reardon and Swinnen 2020).

From a consumer perspective, reduced income may lead to less purchasing power for food, particularly among the vulnerable. Phone surveys from multiple countries suggest widespread income reductions in both urban and rural areas (e.g., Amare et al. 2020; Josephson, Kilic, and Michler 2020; Mobarak and Vernot 2020; Nestour, Mbaye, and Moscoviz 2020; Wieser et al. 2020). Although lockdowns may have led to income losses, the severity of these losses remains largely unknown, and lockdowns or restrictions on movement have varied substantially by country.

Related to household incomes, evidence from Addis Ababa is no different from evidence in other parts of the world. In a phone survey conducted in early May, 58% of respondents stated that their incomes had fallen relative to their standard income at that time of the year (Hirvonen, Abate, and de Brauw 2020); in July, that percentage remained high at 64% (de Brauw, Hirvonen, and Abate 2020). Phone survey respondents also suggest the most common shock to their household has been either unemployment or a loss of income (de Brauw, Hirvonen, and Abate 2020; Hirvonen, Abate, and de Brauw 2020; Abate, de Brauw, and Hirvonen 2020b).

We use data collected from a representative sample households in Addis Ababa to study how the COVID‐19 shock has affected their food consumption. We collected baseline data for a randomized control trial (RCT) in August and September 2019, and collected endline data for the RCT in January and February 2020. When the COVID‐19 pandemic and associated movement restrictions occurred soon thereafter, we began collecting monthly phone surveys between May and August. In the August survey, we collected a third round of food consumption data. Relative to the September 2019 survey, we find no evidence of a decline in food security by August 2020 among the 577 households for which we have detailed food consumption data for both survey rounds. Moreover, the distribution of food consumption is nearly unchanged; if anything, household members consume more food post‐pandemic than they were before. This finding is at odds with the subjective evidence described above about income declines and appears in contrast with much of the international narrative related to COVID‐19 and food insecurity.

To begin to reconcile these differences, we first explore heterogeneity in the composition of food consumption. We find consumption of staples has risen, whereas consumption of legumes and vegetables have fallen. Meanwhile, fruit and animal source food consumption remained the same on average, suggesting indirectly that several value chains, even of perishable foods, continued to function well. However, we find almost no evidence that those reporting reduced incomes are more likely to reduce consumption of more expensive foods, suggesting that the changes in demand relate to changing prices rather than declining demand among some households.

The evidence in the paper therefore suggests that relying on subjective questions about income changes found in most phone surveys to model changes in food security will overstate food security concerns. Further, the evidence here is consistent with phone survey evidence that value chains have largely been resilient to the pandemic (Tesfaye, Habte, and Minten 2020; Hirvonen et al. 2020). In other words, in facing the pandemic shock, households have found ways to smooth food consumption, and food availability appears to be relatively high.

The results are therefore more suggestive that movement restrictions occurring early in the pandemic acted more like a temporary shock than a permanent one. After an adjustment period, people began to find ways to continue to (or find) work, they found ways to ensure that value chains continued to provide food to markets, and to the extent that they have faced income shocks, they have found ways to smooth those shocks. After adjusting to the new equilibrium with less movement and less personal contact, markets began to work smoothly again.

To make this argument, the paper proceeds as follows. The next section provides more background about the Ethiopian response to COVID‐19, followed by a more detailed data description. We then discuss the results on self‐reported income changes and in the same section contrast these measures with more objective reports of food security and food consumption. We then provide suggestive evidence on explanations for the disconnect between the food consumption data and reports of reduced income. The final section describes implications of our results, including those related to subjective questions about income, and suggests further research on food value chains.

## Context

### Addis Ababa

In 2016, the estimated population of Addis Ababa was 3.8 million (Central Statistical Agency 2018b), out of which 16.8% had consumption levels below the national poverty line (Central Statistical Agency 2018a). Virtually all households have access to electricity, more than 90% are connected to piped water, and more than half have access to improved sanitation (World Bank 2020). About 44% of households in Addis Ababa are headed by women. The average household size is four members (Central Statistical Agency 2018b).

Data from the 2016 Demographic and Health Survey show a co‐existence of under‐ and over‐nutrition in Addis Ababa (Central Statistical Agency and ICF 2016). Nearly 15% of children under five years of age in the city are chronically undernourished (stunted; short for their age). Meanwhile, 13% of women and 18% of men between the ages of fifteen and forty nine years are thin with a body‐mass index (BMI) of less than 18.5, even as 29% of women and 20% of men are overweight or obese with a BMI above twenty five.

According to the 2018 Urban Employment Unemployment Survey of the Central Statistical Agency (CSA), 20% of the working age population in Addis Ababa are unemployed, and 30% of the employed population are self‐employed (Central Statistical Agency 2018b). In terms of sector of employment, 20% work in wholesale and retail trade, 13% in manufacturing, 8% in construction, and 5% in accommodation and food service activities (Central Statistical Agency 2018b). About 10% work for other households as, for example, servants or guards. Finally, 9% of the working age population in Addis Ababa work in the informal sector.^1^

### COVID‐19 Policy Measures in Ethiopia

The first COVID‐19 case was confirmed in Ethiopia on March 13, 2020. By August 30, more than 890,929 laboratory tests had been conducted, out of which 51,122 were positive (6% of all tests). More than 60% of the these positive tests have been in the capital, Addis Ababa. By August 30, there had been 793 deaths in Ethiopia attributed to the virus (Ministry of Health and Ethiopian Public Health Institute 2020).

The first policy measures to limit the spread of COVID‐19 in Ethiopia were declared on March 16, just three days after the first confirmed case. The government of Ethiopia closed schools, banned all public gatherings and sporting activities, and encouraged physical distancing. Travelers from abroad were put into a mandatory quarantine, bars were closed until further notice, and travel through land borders was prohibited. Several regional governments imposed restrictions on public transportation and other vehicle movement between cities and rural areas.

A federal level State of Emergency was declared on April 8. Land borders were closed, except for cargo. Facemasks became compulsory in public spaces. Restrictions on cross‐country public transportation and city transportation were also declared; for example, the carrying capacity of public transportation providers was limited to half of their normal capacity. The government also prohibited employers from laying off their workers and property owners from evicting their tenants or increasing rents during the State of Emergency. Some administrative regions took even stricter measures by closing restaurants and limiting movement between rural and urban areas. Adherence to these measures and other recommended virus prevention practices were reportedly high (de Brauw, Hirvonen, and Abate 2020; Hirvonen, Abate, and de Brauw 2020; Abate, de Brauw, and Hirvonen 2020b). However, in contrast to some other countries in the region, Ethiopia never went into a full lockdown that severely restricted movement, imposed curfews, or fully closed all borders. A full lockdown was not imposed to protect the economically most vulnerable segments of the population (France‐24 2020). As of July, movement across regional states was allowed, and humanitarian organizations were permitted to operate without restrictions (United Nations Office for the Coordination of Humanitarian Affairs 2020). Official inflation estimates suggest that food prices have risen during the pandemic but not at an unusually fast rate relative to pre‐pandemic inflation rates (see Hirvonen 2020).

The main social protection response to COVID‐19 in Ethiopia has come through the Productive Safety Net Programme that operates in urban and rural areas.^2^ Launched in 2005 in food insecure rural areas and in 2017 in selected urban areas, PSNP is managed by the Government of Ethiopia and is mostly funded by a consortium of international organizations and development partners. The PSNP provides monthly cash or food transfers against labor‐intensive public works that build community assets. Eligible households with limited labor capacity receive unconditional cash transfers. Due to the pandemic, the public works requirement was waived, and thus all beneficiaries now are receiving unconditional transfers. At the beginning of the pandemic, beneficiaries also received three months of payments in advance (Gentilini et al. 2020). In addition to the PSNP, several smaller scale initiatives have been launched to support poor and vulnerable households, including food banks set up by city administrations, community support, and NGO programs.

## Data

Our COVID‐19 telephone survey in Addis Ababa builds on an earlier IFPRI‐led randomized controlled trial testing the effectiveness of video‐based behavioral change communication to increase fruit and vegetable consumption in the city (Abate et al. 2019).^3^ The baseline (or pre‐intervention) survey for this project was administered in August and September 2019 with an endline (or post‐intervention) survey in January and February 2020—approximately one month before the first confirmed COVID‐19 cases in Ethiopia. The phone surveys were administered in early May, June, July, and August. Table 1 shows the dates and the sample sizes for each survey round as well as the type of food consumption module administered in the survey. Below we provide more details about the in‐person and phone surveys.

**Table 1:** Survey Times, Sample Sizes and the Type of Food Consumption Module, by Survey Round

Survey round	Dates	Sample size	Food consumption module
In‐person survey #1	August 21–September 20, 2019	930	Food item level
In‐person survey #2	January 24–February 11, 2020	895	Food item level
Phone survey #1	May 01–May 05, 2020	600	Food group level
Phone survey #2	May 30–June 06, 2020	589	Food group level
Phone survey #3	June 27–July 04, 2020	584	Food group level
Phone survey #4	August 01–August 08, 2020	577	Food item level

*Note*: The phone surveys were based on a random subsample of the sample used in the in‐person surveys.

### In‐Person Surveys

In designing these surveys, we adopted a stratified random sampling approach based on household welfare levels to ensure a balanced sample between wealthy and less wealthy neighborhoods, and between poor and rich households.^4^ The baseline survey was administered between August and September in 2019 and covered 930 households. The endline survey took place between January and February 2020, and 895 households were interviewed (96% of the baseline sample). The January and February 2020 survey instrument collected detailed information about household demographics, income sources, asset levels, food consumption, and food security. We use the information collected about asset levels to construct a pre‐pandemic asset index by applying a principal components method (see the online supplementary appendix) and use this index to contrast the food security outcomes between wealthy and less‐wealthy households.

We do not use the consumption data from the January‐February survey in the main analysis as a comparison for two reasons.^5^ First, as mentioned, it acted as an endline for an RCT, and so a treatment effect could have affected some types of food consumption. But perhaps more importantly, we cross‐randomized a survey experiment to better understand the impacts of telescoping on food consumption measures; given that we find what appears to be substantial telescoping bias (Abate et al. 2020a), the distribution of consumption in the February survey is affected by the two experiments.^6^

### Phone Surveys

To understand how the COVID‐19 crisis is affecting households in Addis Ababa, we administered a series of phone surveys with a subsample of households that participated in the in‐person surveys.^7^ The phone surveys used phone numbers for members of the sample for the survey conducted in January and February 2020. Phone numbers were collected from 99% (887 households) of the 895 sample households that took part in the February survey. Out of these households, we drew a subsample of 600 households. The first phone survey was administered in early May, and follow‐up surveys in early June, July, and August.^8^ Attrition rates remained relatively small. In our final phone survey in August, we managed to reach 577 households out of the 600, implying an attrition rate of 3.8%.

To minimize the risk of response bias (Dabalen et al. 2016; Lau et al. 2019), we used sample stratification and replacement techniques in the first phone survey. We first split the sample into deciles according to household asset holdings, and then randomly selected sixty households from each decile (600 households in total). If the enumerators were unable to reach a selected household after five attempts, it was replaced with another randomly selected household in the same asset decile. Because some households could not be reached in the initial sample, they were replaced with another randomly selected household in the same decile. In total, forty six (or 7.7%) of the initial 600 households could not be reached in the first phone survey administered in early May and were replaced. Apart from one household, all households that were reached agreed to take part in the survey.

Based on key household characteristics (sex, age, and education level of household head; household size and asset levels; household dietary diversity indicators) measured in January and February 2020, the final subsample that took part in the first phone survey is very similar to those households that took part in the pre‐pandemic face‐to‐face survey but were not interviewed in the phone survey in May 2020.^9^ Table B4 in the online supplemental appendix shows the demographic composition of the sample households remained similar before and during the pandemic in 2020. Although changes in dependency ratios are not statistically different from zero, we observe a small decrease in household size between September 2019 and February 2020 rounds.^10^

The first three phone survey instruments focused on questions about food and nutrition security and self‐reported changes in income sources and levels.^11^ In August survey, we replaced this questionnaire with a comprehensive household food consumption module, identical to the one administered in the in‐person surveys in September 2019 and February 2020.

## Results

### Self‐Reported Income Changes

**Figure 1:**
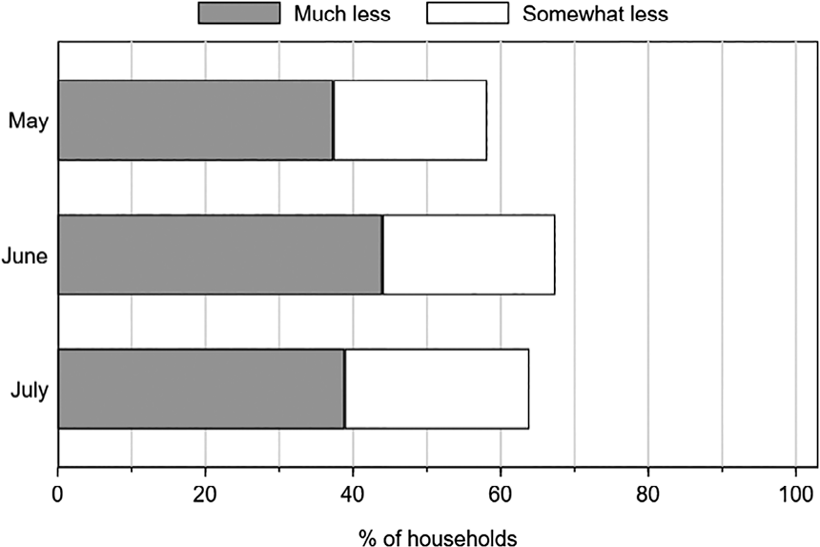
Self‐reported changes in income levels in the past month compared to usual incomes, by survey round. *Note*: N = 600 households in May; 589 households in June and 584 in July

In the May, June, and July phone surveys we asked respondents to compare incomes they received in the last month to the incomes they usually receive at this time of the year. Figure 1 shows that in each survey round, over 50% of respondents stated their household incomes were lower or much lower than usual. For example, in July 64% of respondents reported their incomes were lower in the past month than usual.

**Figure 2:**
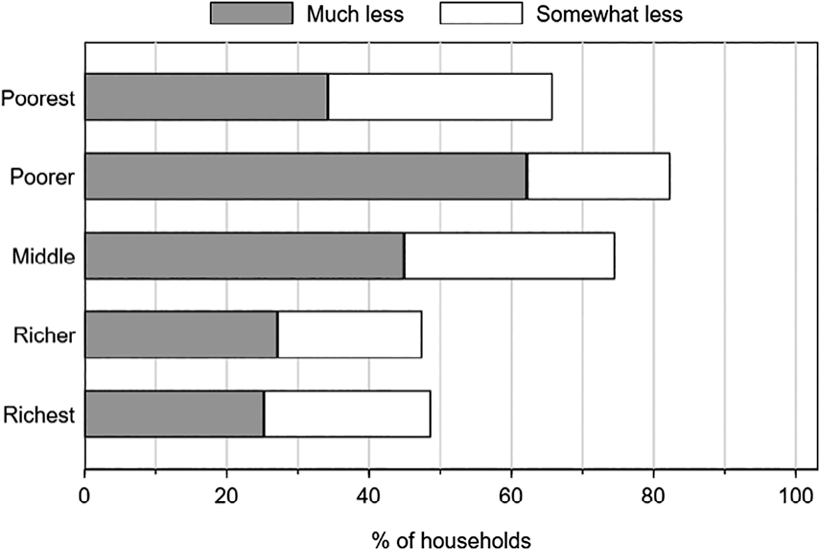
Self‐reported changes in income levels in the past month compared to usual incomes, by asset levels (July survey only). *Note*: N = 584 households. The wealth quantile grouping is based on a wealth index constructed using a principal components method based on household asset ownership using data collected in the February 2020 survey.

Using the pre‐pandemic asset index described in the previous section, we can further assess how these responses varied across wealth quintiles. In the July survey, poorer households are considerably more likely to report income losses than richer households (figure 2). Whereas more than 60% to 80% of the poorest two quintiles reported income losses, less than 50% of the richest two quintiles did so.

**Figure 3:**
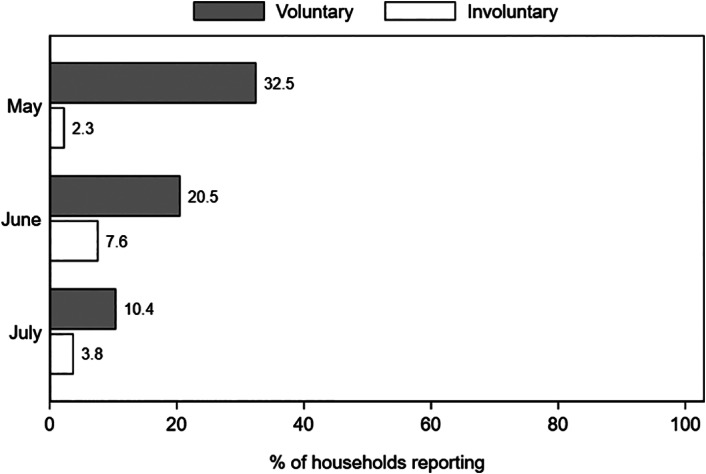
Self‐reported voluntary and involuntary job losses in the previous month, by survey round. *Note*: N = 600 households in May; 589 households in June and 584 in July. Voluntary job loss refers to a situation where one or more household member quit their job voluntarily and involuntary to a situation where one or more household member's written or verbal contract was terminated by the employer.

We also asked respondents whether there were any changes in the employment status of the household members in the last thirty days prior to the interview. We see that job losses during the pandemic were high but mostly voluntary in nature where a household member him or herself terminated the contract (figure 3). Ethiopia's private sector is characterized by very high job turnover (Blattman and Dercon 2018; Abebe et al. 2020; Söderbom, Shiferaw, and Alemu 2020).^12^ Considering this turnover, it is not clear the pandemic has led to higher than usual unemployment rates in Addis Ababa.^13^

### Food Security Indicators

Given the reported decline in incomes, we next explore whether food security declined among sample households. If income declines were substantial, we might expect to observe a decline in dietary diversity. All survey rounds permit us to construct a Household Dietary Diversity Score (HDDS) in which consumed food items are grouped into twelve food groups (Swindale and Bilinsky 2006).^14^ Assigning a value of one for each food group that the household consumed from and summing, we can construct the HDDS in which higher scores indicate a better household food security situation. HDDS is a widely used food security indicator and previous work has found it to be highly correlated with caloric availability (Hoddinott and Yohannes 2002) and nutrient adequacy (Mekonnen et al. 2020). In May, June, and July rounds, we administered the standard HDDS module that asks households whether they consumed from a given food group. For the September, February, and August rounds, we use data from a detailed item‐level food consumption module to construct the HDDS.

**Figure 4:**
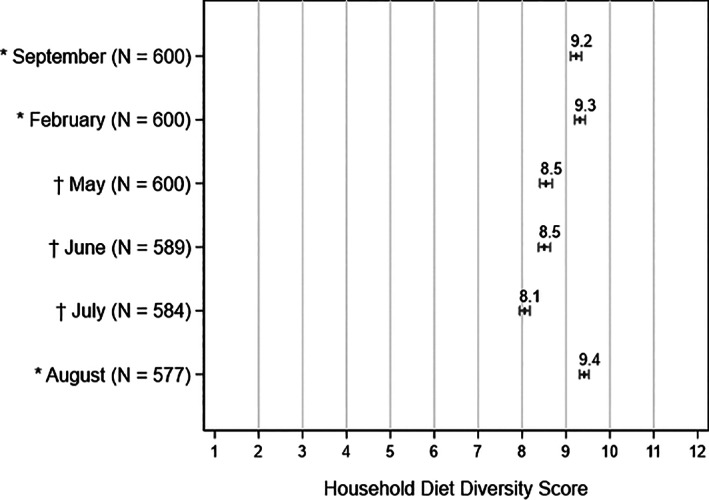
Mean household diet diversity score, by survey round. *Note*: Capped lines represent 95% confidence intervals. * = Estimates based on data from a comprehensive item‐level food consumption module. † = Estimates based on data from a less detailed, food group‐level food consumption module.

Figure 4 shows the average HDDS for each survey round.^15^ We observe that the average HDDS initially fell during the first phone survey rounds, which could be due to the adverse impact of the COVID‐19 pandemic or because of the change in the survey methodology from detailed food consumption module to a series of yes/no questions about consumption from food groups (Table 1). The average HDDS in the sample was 9.2 in September and 9.3 in February. In the May and June surveys, the average HDDS was 8.5, and fell to 8.1 in the July survey, most likely because the recall period coincided with an Orthodox fasting period during which Orthodox households abstain from animal source foods. In August, when we use the same survey module as in the in‐person surveys, we obtain an average HDDS of 9.4, very similar to the average estimated before the pandemic.

Of note is that even the lowest mean HDDS recorded during the pandemic is well above average scores recorded in other surveys administered before the pandemic. Using the nationally representative 2015/2016 Living Standards Measurement Study‐Integrated Surveys on Agriculture (LSMS‐ISA) survey for Ethiopia, Mekonnen et al. (2020) estimate an average HDDS of 6.2 for rural areas and 7.9 for urban areas. Moreover, in July 2018, the mean household diet diversity score in chronically food insecure areas supported by the rural PSNP was less than five food groups (Berhane et al. 2019a, Berhane et al. 2019b). These numbers suggest that the average food security situation in Addis Ababa at the height of the pandemic was considerably better than in other areas before the pandemic.

**Figure 5:**
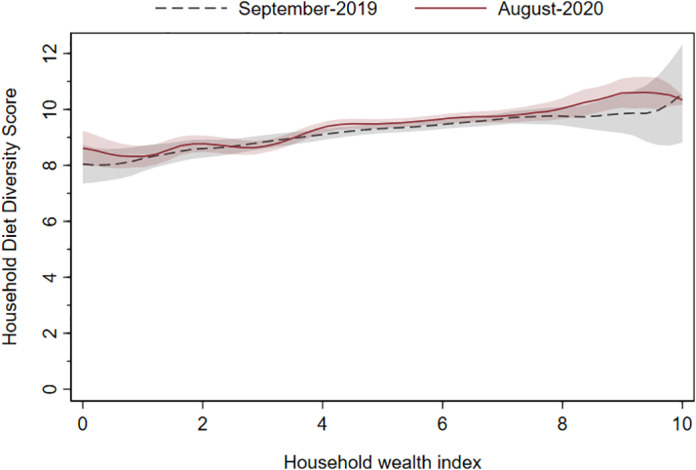
Household wealth and household dietary diversity score. *Note*: Local polynomial regression. N = 577 households in both rounds. The shaded areas represent 95% confidence intervals. The recall period is last seven days. The wealth index (horizontal axis) is constructed using a principal components method based on household asset ownership using data collected in the January and February 2020 Addis Ababa food consumption survey. The wealth index has been scaled to 0–10.

In figure 5, we use data from September 2019 and August 2020 surveys, and estimate local polynomial regressions to examine HDDS across pre‐pandemic asset levels. Although richer households have higher HDDS than poorer households in both rounds, the two regression lines lie on top of each other. Statistically, this finding implies we cannot detect a difference in HDDS between these two rounds. At least in relatively crude terms, diets do not appear to have been affected by the COVID‐19 pandemic at any wealth level.

Next, we explore how the subjective income shocks are correlated with HDDS. Using data from May, June, and July survey rounds (for which we asked about the subjective income shocks), we use a household fixed effect model to regress HDDS on a binary household income shock variable.^16^ Table 2 reports the results for household fixed effect model without (column 1) and with (column 2) survey round fixed effects. In both regression models, the coefficient on the income shock variable is close to zero and statistically insignificant, indicating the self‐reported income shocks do not appear associated with changes in HDDS.

**Table 2:** Association between Self‐Reported Income Shock and Household Diet Diversity Score, Household Fixed Effects Regression

	(1)	(2)
Income shock	−0.053 (0.095)	−0.031 (0.091)
Household fixed effects?	Yes	Yes
Survey round fixed effects?	No	Yes
Observations	1,773	1,773

*Note*: Dependent variable is household diet diversity score (HDDS). Data are based on phone survey data collected in May, June, and July 2020. The income shock variable obtains value 1 if households reports to have received "Much Less" or "Less" income in the past month prior to the survey round, and zero otherwise. Standard errors clustered at the level of fixed effect (i.e., household level) and reported in parentheses.

### Food Consumption Outcomes

At this point, we have established that although more than half of households have been reporting reduced incomes relative to this time of year, household dietary diversity—a widely used measure of food security—has not suffered. However, households could have had to reduce the amount of food consumed over time, or they could have changed the composition of consumption without changing the number of food groups consumed. We next explore these possibilities.

Both in‐person surveys and the August phone survey collected detailed information on households' food consumption over the seven days prior to the survey interview. The quantity of each food items consumed was reported in standard units (grams, kg, liter, etc.). We valued the amounts of food consumed in Ethiopian birr using monthly retail price data for Addis Ababa provided by the CSA. To adjust for inflation, we used CSA retail price data from September 2019 in all survey rounds. We also converted amounts consumed into calories using food composition tables provided by the Ethiopian Public Health Institute (Ethiopian Public Health Institute 1981) with estimates of item‐specific edible portions obtained from U.S. Department of Agriculture (2013).

**Figure 6:**
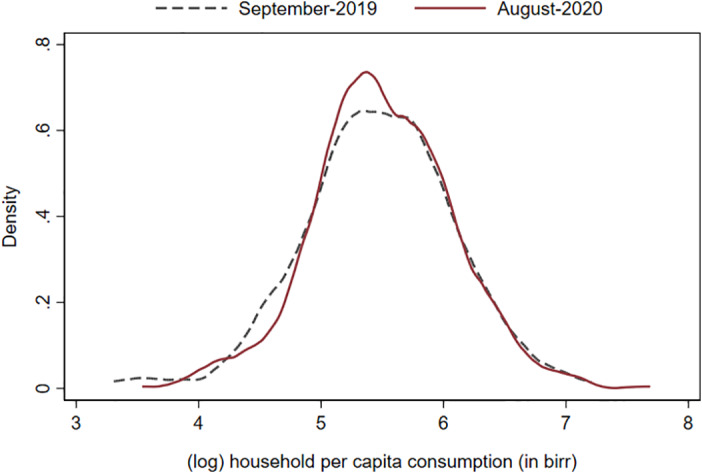
Household per capita consumption (in birr) distributions in September 2019 and August 2020. N = 577 households in both rounds

We first plot probability distribution functions (PDFs) of the value of log household consumption per capita among panel households in the September 2019 and August 2020 survey rounds (figure 6).^17^ If we expect food consumption would have dropped due to the pandemic, the August 2020 PDF should be shifted to the left of the September 2019 PDF. If the only effects were among poorer households, the shift should occur at the bottom end of the distribution. We find no evidence of either type of shift. If anything, the August 2020 PDF is shifted to the right of the September 2019 PDF, suggesting that households on the poorer end of the distribution are spending more on food during the pandemic than they did before. On average, in fact, the value of food consumption increased by 2% between the September 2019 and August 2020 surveys, although the difference is not statistically different from zero (*p* = 0.62).

**Figure 7:**
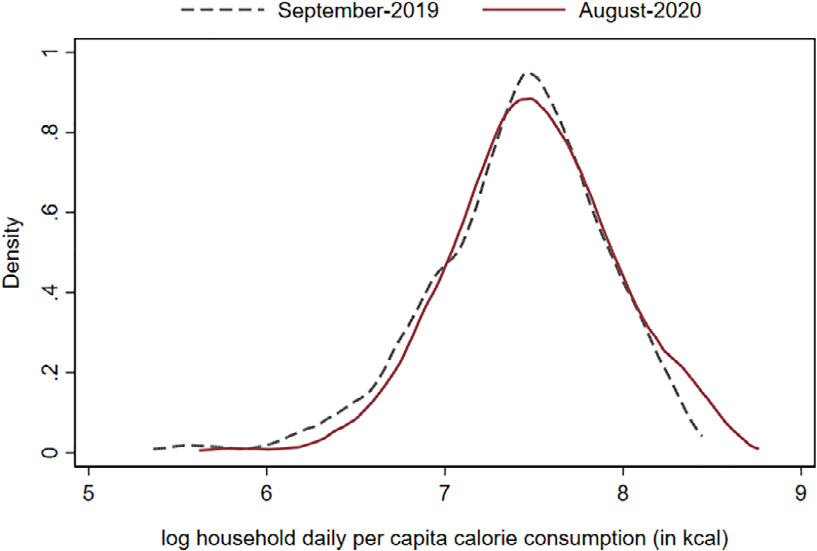
Household per capita consumption (in kcal) distributions in September 2019 and August 2020. N = 577 households in both rounds

This pattern is confirmed when PDFs are plotted for log calories consumed per capita rather than the value of consumption (figure 7). In August 2020, the entire PDF has shifted to the right of that in the September 2019 survey, and calories per capita had increased by 9%, on average. Clearly, there is no evidence that food consumption has fallen among in our sample, regardless of total food consumption level.

There are a few reasons food consumption may have remained resilient overall during the pandemic. First, a small share of food is eaten away from home; in the September 2019 survey, we placed substantial emphasis on collecting improved data on food consumed away from home, and we find that it only represents 7% of food expenditures (Wolle et al. 2020). Unlike countries where consumers eat a substantial amount away from home, so long as value chains were resilient, there would be no reason to substantially change food distribution patterns. Second, note that consumers may have substituted non‐food consumption for food consumption, especially as some outlets for spending (e.g., bars, cinemas) were closed during the pandemic.

Even if food consumption has not declined or has even risen for most groups, the composition of consumption could have changed. For example, in the July phone survey, we asked if people were avoiding any foods during the pandemic, and 59% stated they were avoiding raw vegetables, whereas 61% stated they were avoiding raw meat.^18^ In table 3, we split food consumption into seven categories, combining some of the categories in the HDDS: staples (which includes cereals, roots, and tubers); legumes and nuts; vegetables; fruits; meat and eggs (including fish); dairy products; and other foods (which includes oil, sugar, and miscellaneous foods). We find that there has been a change in the composition of food consumption. Consumption of staples has increased by 11% on average, whereas consumption of legumes and nuts and vegetables have declined by 16% and 19% on average, respectively. Whereas there are differences for the other categories, they are relatively small in magnitude and not statistically different from zero. Because prices are held constant, these findings suggest there has been some shift from legumes and vegetables to staples (as nuts are a small share of consumption).

**Table 3:** Mean Weekly Per Capita Consumption in Birr, by Food Group

Food group	September 2019	August 2020	Difference	Difference in %‐terms
Staples	81.48	90.8	9.32***	11%
Legumes and nuts	21.38	18.00	−3.38***	−16%
Vegetables	57.39	46.32	−11.07***	−19%
Fruit	17.33	19.45	2.12	12%
Meat and eggs	60.37	67.65	7.28	12%
Dairy products	12.08	10.33	−1.75	−14%
All other foods	35.31	37.42	2.11	6%
**Total**	**285.34**	**289.97**	**4.63**	**2%**

*Note*: N = 577 households in both rounds. Difference in means between the groups tested with a t‐test (null‐hypothesis: difference in means = 0). Statistical significance denoted with *p < 0.1; **p < 0.05; ***p < 0.01.

We next examine the composition of average calories consumed daily per capita, by food group (table 4). Similar to table 3, we find an increase in staple calories consumed, whereas the calories per capita of legumes and nuts and vegetables both decline. We also find a statistically significant increase in per capita consumption of fruit and a decline in caloric consumption of all other foods.

**Table 4:** Mean Daily Per Capita Calorie Consumption, by Food Group

Food group	September 2019	August 2020	Difference	Difference in %‐terms
Staples	1,025.9	1,263.6	237.7***	23%
Legumes and nuts	160.5	130.4	−30.1***	−19%
Vegetables	114.7	85.3	−29.4***	−26%
Fruit	33.2	39.8	6.6**	20%
Meat and eggs	51.0	54.4	3.4	7%
Dairy products	33.1	37.9	4.8	15%
All other foods	410.0	387.1	−22.9*	−6%
**Total**	**1,828.4**	**1,998.5**	170.1***	**9%**

*Note*: N = 577 households in both rounds. Difference in means between the groups tested with a t‐test (null‐hypothesis: difference in means = 0). Statistical significance denoted with *p < 0.10, **p < 0.05, ***p < 0.01.

The combination of these two results suggests there has been a shift from some relatively expensive calories (e.g., vegetables) to cheaper ones (staples). Even within categories, the same appears to be true; for example, because the calories of fruit consumed rose more than the value of fruit consumed, there must have been a shift from slightly more expensive fruit, in terms of calories, to less expensive ones. However, note again that the total food budget did not change; therefore, changes are happening along the intensive rather than the extensive margin.

There are several potential explanations for the overall patterns of results above; we can rule some of them out with the data. First, people could be avoiding certain types of foods. Recall that in the June and July surveys, about 60% of respondents suggested they were avoiding uncooked vegetables due to COVID‐19 risk, and between 60% and 65% of respondents were avoiding uncooked meat for the same reason (de Brauw, Hirvonen, and Abate 2020; Abate, de Brauw, and Hirvonen 2020b). This taboo could have affected overall vegetable consumption. However, when we split the sample by households that say they are avoiding uncooked vegetables versus those that are not, we find that per capita consumption of vegetables is actually higher in households avoiding uncooked vegetables than in other households. Because meat consumption did not decline in general, it seems that food taboos due to COVID‐19 did not affect demand for specific classes of foods.

Alternatively, in line with Bennett's (1941) law, the decline in consumption of legumes and vegetables could be concentrated among households that had larger negative income shocks related to COVID‐19. In other words, the summary statistics in tables 3 and 4 may mask important heterogeneity, in which households exposed to income shocks shifted their diets toward staples and other households maintained their diets as in the previous year. To examine this hypothesis, we split the sample by whether households reported having “much less” or “less” income in the July phone survey round than usual, relative to households that reported no change or a positive change. We choose the July survey as it was closest in time to the food consumption recall period. We then measure the difference between the September 2019 and August 2020 surveys, and report whether the difference in differences is statistically different from zero. Table 5 reports the results of this difference in differences exercise. The bottom row shows that households that did not report an income shock saw their per capita consumption levels increase by 6.2% (or 18 birr) between the two survey rounds. In contrast, the per capita consumption level of households reporting an income shock decreased only by 1.0% (or 2.9 birr). However, neither difference is statistically different from zero.

**Table 5:** Change in Mean Weekly Per Capita Consumption in Birr between September 2019 and August 2020, by Food Group and Income Loss Status in July

	Income loss	No income loss	Difference in differences
Staples	4.69	17.30	12.61**
Legumes and nuts	−3.35	−3.43	−0.08
Vegetables	−13.87	−6.25	7.62*
Fruit	0.51	4.91	4.40
Meat and eggs	8.59	5.02	−3.57
Dairy products	−1.31	−2.48	−1.17
All other foods	1.80	2.63	0.83
**Total**	**−2.94**	**17.70**	**20.64**

*Note*: N = 577 households in both rounds. Difference in means between the groups tested with a t‐test (null‐hypothesis: difference in means = 0). Statistical significance denoted with *p < 0.10, **p < 0.05. Household incurred an income loss if it reported to have received “Much less” or “Less” income than usual in the month preceding the July survey (see Figure 1).

Second, relative prices for different types of foods could have changed; for example, if vegetables and legumes became more expensive either for reasons related to COVID‐19 or for other reasons, households may have reduced their demand for those foods and instead consumed cheaper staples (alternatively, prices for staples could have dropped). We therefore conduct analysis of available prices, which lends at least partial support to this hypothesis (Appendix A). Prices of staples and legumes in Addis Ababa increased by about 20% between September 2019 and August 2020. Monthly price increases were remarkably steady, with no clear structural break when the pandemic began. Moreover, the price increases in the two food groups are consistent with overall food price inflation, which has been around 20% in pre‐pandemic years (Hirvonen 2020). In contrast, the prices of vegetables increased by 56% since March 2020, or the onset of the pandemic.^19^ Thus, the rapid increases in vegetable prices could have caused households to shift their food consumption from vegetables to starchy staples.

Whereas some results are suggestive that diets worsened among those reporting income losses, others are not. For example, households reporting no income loss increase the value of their staple food consumption more than those reporting income losses; this difference is statistically significant. Whereas households reporting income losses have a larger decline in the value of vegetable consumption, they increase meat and eggs consumption more than those reporting no income loss and have a smaller decline in the value of dairy consumption, though neither of the latter differences are statistically significant. In sum, these differences are only slightly suggestive of the patterns that we would have expected to observe if households with income losses had relatively worse diets as a result of the pandemic.

When we reconstruct the table using calories instead of value (Table 6), we find an overall gain in per capita calorie consumption by both groups, and the only difference significant at the 5% level is in staples consumption; reported per capita staples consumption among households with no income loss increased by 346 calories per capita, versus 174 calories per capita among those reporting income losses. The difference‐in‐differences result for calories from vegetables is only significant at the 10% level, again suggesting that the difference is not that large.

**Table 6:** Change in Mean Daily per Capita Calorie Consumption (in Kcal) between September 2019 and August 2020, by Food Group and Income Loss Status in July

	Income loss	No income loss	Difference in differences
Staples	174.4	346.8	172.4**
Legumes and nuts	−32.3	−26.2	6.1
Vegetables	−34.1	−21.1	13.0*
Fruit	4.1	10.9	6.8
Meat and eggs	3.4	3.2	−0.2
Dairy products	5.6	3.2	2.4
All other foods	−38.9	4.6	43.5*
**Total**	**82.2**	**321.5**	239.3**

*Note*: N = 577 households in both rounds. Difference in means between the groups tested with a t‐test (null‐hypothesis: difference in means = 0). Statistical significance denoted with *p < 0.10, **p < 0.05. Household incurred an income loss if it reported to have received “Much less” or “Less” income than usual in the month preceding the July survey (see Figure 1).

In the online supplemental appendix (Tables B6 and B7), we disaggregate changes in per capita consumption levels by using households' job loss status (see figure 3) instead of their loss in income. As before, we find no clear evidence that the job losses reported by the households resulted in major changes in household consumption patterns between September 2019 and August 2020.

In sum, there is no clear pattern of heterogeneity suggesting there is a class of households that due to a loss of income or a job shift toward staples away from more expensive types of foods (fruit, vegetables, animal source foods). Regardless of categorization it seems that all households increased their consumption of staples relative to other types of foods. This pattern is much more suggestive of changes in relative prices than in heterogeneity of demand changes related to changes in income, a hypothesis that is partially confirmed by our analysis of retail prices during the study period (see Appendix A). Our results are limited in that we cannot disentangle to what extent government or non‐governmental programs (e.g., the PSNP) may have helped maintain HDDS or calorie consumption per capita.^20^ Moreover, we cannot definitively state the nutritional implications of the results; the decline in reported household vegetable consumption might be considered concerning, but we have household level rather than individual level data, and moreover, other more nutrient dense foods have not declined or have potentially increased as a share of the diet.

## Conclusions

We use panel data collected before and after the COVID‐19 pandemic began to assess whether and how food security has changed among representative sample households in Addis Ababa, the capital of the second largest country in Africa. Five months into the pandemic, we find that a standard food security indicator (HDDS) has not changed from September 2019, and if anything, we find that an increase in calories consumed in a seven‐day recall. These results therefore suggest that food security situation in Addis Ababa is largely unchanged, even at the lower end of the distribution. This finding is in contrast both with evidence from subjective income measures from these households, as well as with concerns about increasing food insecurity that has been suggested by international humanitarian organizations (e.g., World Food Programme ).

Though overall food consumption does not change, we do find shifts in the pattern of food consumption toward staples and away from vegetables. This shift can be at least partially explained by changes in relative prices. Still, there are potential explanations that we cannot rule out. First, the shock induced by the pandemic could have been really short term. Along these lines, when we first called the panel households in May 2020, consumers and markets may have already adjusted to the new equilibrium with less movement and less personal contact. Second, households could have maintained food consumption by cutting back on non‐food consumption or financing food consumption through savings or taking on debt. Because some services were no longer available, some of the money they would have previously spent on entertainment or other no longer available services could have been instead spent on food.

Although households in Addis Ababa are better off on average than households in rural and other urban areas of Ethiopia, the virus has been spreading faster in the capital, possibly because of the higher population density. Measures to contain the virus also have stronger effects on urban residents because their livelihoods are more likely to be in sectors that are more adversely affected by social distancing policies and travel bans. Therefore, the World Bank predicts that the poverty impacts of the pandemic will be focused on urban areas (Nguyen et al. 2020). Moreover, possible disruptions to food value chains are more detrimental to urban households because they typically do not grow their own food. Despite these predicted challenges, our findings suggest household food consumption in Addis Ababa have been highly resilient during the COVID‐19 pandemic. Meanwhile, understanding the pandemic impacts in rural areas of Ethiopia has been hampered by the fact that only 40% of the rural households have an access to a phone and that phone owning households are on average wealthier and more educated with better access to basic amenities such as electricity, water, and sanitation (Wieser et al. 2020). Although we are not aware of surveys reporting on the collection of detailed consumption data in rural Ethiopia, financial diary data collected from neighboring Kenya show food expenditures among rural households remained at pre‐pandemic levels during the first weeks of the pandemic (Janssens et al. 2020).

Although the evidence in this paper is exclusively descriptive, the results at least cast doubt about the value of subjective questions about income in phone surveys. Based on a scan of the RECOVR website hosted by Innovations for Poverty Action and data made public by the Living Standards Measurement Surveys team at the World Bank, post COVID‐19 phone surveys are primarily using subjective income shocks to study the effects of the pandemic on household well‐being. The response options to these questions are typically qualitative, for example: “incomes were much lower”; “somewhat lower”; “same”; “higher”; “much higher.” Although these responses provide some idea of the direction of income trends, they are difficult to interpret when it comes to magnitude of the income loss (De Weerdt 2008). Apart from genuine differences in income changes across households, variation in responses can also arise from differences in interpretation of the response option thresholds, for example, “much lower” versus “somewhat lower,” or because some respondents are not willing to truthfully answer questions about their incomes. Moreover, despite the retrospective nature of these questions, responses may also be affected by expectations about future income streams amid the widespread uncertainty during the pandemic (Doss, McPeak, and Barrett 2008; Jolliffe, Seff, and De La Fuente 2018). The results in this paper suggest these measures are misleading at best, wrong at worst, and may seriously overexaggerate the welfare and poverty impacts of the ongoing pandemic. Therefore, we suggest more collection of food consumption data, which should be less vulnerable to this criticism as it asks about quantities consumed in the same way across survey rounds.

Our results also provide indirect evidence about the effectiveness of food value chains connecting Addis Ababa. Although we cannot make definitive statements about commodity‐specific value chains, the fact that a representative sample of households are consuming more food, in caloric terms, than they had before the crisis suggests most food value chains have been resilient to the shock associated with the pandemic. Several factors, some specific to Ethiopia, may have helped food value chains continue to function well during the pandemic. First, due to a lack of cold chains, perishables (fruit, vegetables, animal source foods) are produced nearby. Second, food away from home is not (yet) a large portion of the Ethiopian diet, so value chains to restaurants did not have to substantially reorganize themselves as demand from restaurants shrank. Third, although food imports play a role in the Ethiopian diet, in value terms, over half of imports are composed of wheat, palm oil, and sugar; therefore, problems with imports would largely only affect staples or “all other foods” in our formulation, and we only observe a small decrease in consumption of the latter category. As more detailed price data become available for the period during the pandemic, further research can help us understand the performance of commodity‐specific value chains in Ethiopia. Such analysis can help us better understand factors that might lead value chains to break during a crisis, relative to those that are resilient to shocks.

## Supporting information


**Appendix S1.** Supporting Information.

## Figures and Tables

**Figure A1:**
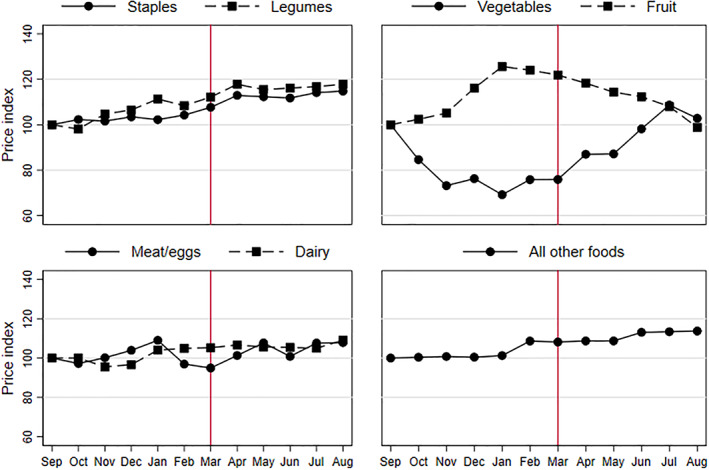
Food group specific monthly price indices (September‐2019 = 100). *Note*: These price indices are weighted averages of item‐level prices for each month between September 2019 and August 2020 where the weights are consumption shares based on our food consumption data collected in September 2019. The vertical solid line marks the onset of the pandemic (March 2020).

## Endnotes

^1^
Central Statistical Agency (2018b) defines informality as "persons who work in an enterprise or business that did not keep book of account, who did not have license and mainly produced for the market were considered to be working in the informal sector".

^2^
Focusing on the areas covered by the rural PSNP, Abay et al. (2020) find the PSNP transfers protected poor households from the negative effects of the pandemic.

^3^
Ethical approval for the in‐person and phone surveys was obtained from IFPRIʼs Institutional Review Board.

^4^
See the online supplementary appendix for more details on the sampling procedure.

^5^
Nevertheless, we illustrate the use of the February 2020 food consumption measure in the two PDFs that make up our main result in the online supplementary appendix (figures ), and they are quite similar to the results shown in the main body of the paper.

^6^
Telescoping occurs when survey respondents either include more distant events in a recall period (forward telescoping) or push recent events further back in time (backwards telescoping).

^7^
This phone study was pre‐registered in the European Economic Association (EEA) and Innovations for Poverty Action (IPA) COVID‐19 registries.

^8^
For participating in the phone surveys, households were given a 100‐birr phone credit (about $3 at the time). The phone credit was mentioned in the introduction to the call in which we also obtained verbal informed consent to participate in the survey. We hypothesize that the relatively high response rate is due to the rapport built up through in person meetings and regular phone calls; the decision to attempt a food consumption survey was based on our low attrition rates in the first three rounds and the hypothesized rapport with households. We extensively pretested the food consumption module used in the August phone survey and only implemented it after we were sure the types of issues that arose were quite similar to issues that arise when fielding in‐person consumption surveys.

^9^
See Table in the online supplemental appendix.

^10^
The most commonly cited reasons for a member leaving the household were “to live with other relatives” (26%), marriage (17%), death (14%), leaving for work (10%), schooling (7%), and divorce (5%).

^11^
We also asked about household knowledge and their behavioral responses to COVID‐19. These are reported in Hirvonen, Abate, and de Brauw, Hirvonen, and Abate (2020); Abate, de Brauw, and Hirvonen (2020b); and de Brauw, Hirvonen, and Abate (2020).

^12^
According to a large‐scale survey of micro and small manufacturing enterprises in 2016/17 (Gebreeyesus et al. 2018), average firm in Addis Ababa hired six new employees over a twelve‐month period. During the same period, four employees voluntarily quit and 0.6 employees were fired. In more than 90% of the cases, the employee quit their job to look for another one with better salary or working conditions.

^13^
Although not specific to Addis Ababa, the high‐frequency phone surveys conducted by the World Bank suggest that urban employment rates plunged at the onset of the pandemic in April but quickly recovered to their pre‐pandemic levels (Ambel et al. 2020).

^14^
The twelve food groups are: cereals; roots or tubers; vegetables; fruits; meat or poultry; eggs; fish and seafood; nuts or pulses; dairy; oil or fats; sugar/honey; and miscellaneous foods.

^15^
Table in the online supplemental appendix shows the mean HDDS values by survey round and household pre‐pandemic asset quintile.

^16^
This variable obtains value =1 if households reports to have received "Much Less" or "Less" income in the past month prior to the survey round, and zero otherwise.

^17^
Using the February 2020 data instead of September 2019 data yields similar findings; see figure in the online supplemental appendix. Following the text, figure illustrates the same graph with calories per capita.

^18^
Raw meat is a local delicacy in many parts of Ethiopia.

^19^
Hirvonen et al. (2020) collect detailed price data at different levels of the vegetable value chain connecting farmers in Central Rift Valley to consumers in Addis Ababa. They find prices of onions and tomatoes increased rapidly during the pandemic, most likely because of the trade disruptions triggered by the pandemic. Of note, the vegetable price index we use give these two vegetables a large weight (53%).

^20^
We did ask about the support households received through these channels. However, in the absence of a random allocation of these programs, it would be difficult use these data to estimate the degree to which PSNP (or other programs) explain the limited changes in HDDS or food consumption levels. Moreover, such analysis would require a different sampling approach (e.g., over‐sampling PSNP households and poor non‐PSNP households) than what we have used here.

## Appendix A Retail Price Analysis

We used CSA's item‐level retail price data for Addis Ababa in September 2019 to value household food consumption. Unfortunately, we were not able to obtain the same comprehensive item‐level retail price data for 2020. Instead, we used the CSA's Country and Regional Level Consumer Price Indices (Central Statistical Agency 2020) to study price trends during the pandemic. This data set is less detailed than the full item‐level data collected by the CSA but does provide some item‐level retail prices for each month. Using the prices reported for Addis Ababa, we manage to obtain prices for forty eight food items out of the 121 items listed in our food consumption module. Together, these forty eight items represent 75% of the total value of the average food basket of our sample in September 2019.

Akin to Laspeyres' index (see Deaton and Tarozzi 2005), we computed a series of food group specific price indices to understand food price dynamics during our study period (September 2019 and August 2020) and during the pandemic that began in March 2020. These price indices are weighted averages of item‐level prices for each month between September 2019 and August 2020 where the weights are consumption shares based on our food consumption data collected in September 2019. We then re‐scale these price indices to 100 for September 2019 permitting us to report the nominal price variations in percentage terms relative to this base period.

Figure A1 provides the results. We see that prices of staples and legumes grew in tandem and were 18% and 20% higher, respectively, in August 2020 as compared to September 2019. The prices of animal sourced foods and “all other foods” also grew relatively steadily during the study period.^21^ In contrast, we see major fluctuations in prices of fruits and vegetables during the year. In line with the vegetable value chain analysis carried out by Hirvonen et al. (2020), the prices of vegetables in Addis Ababa increased by 56% since the onset of the pandemic. Meanwhile, fruit prices declined by 12% during the pandemic. This could be part of normal seasonal fluctuation or due to transportation restrictions that resulted in temporary oversupply of fruit to Addis Ababa. Further research is needed to confirm these conjectures.
