# Supplementary material for: Food Consumption and Food Security during the COVID‐19 Pandemic in Addis Ababa
Source: Am J Agric Econ. 2021 Feb 23;103(3):772–89. doi: 10.1111/ajae.12206 (PMC8013419; doi:10.1111/ajae.12206)
Supplement: Supplementary file 1 — Appendix S1. Supporting Information. [file AJAE-103-772-s001.zip › AJAE MS#20450-Online Supplementary Appendix-de Brauw.pdf]

Online Supplementary Appendix for “Food Consumption and Food Security during the COVID-19 Pandemic in Addis Ababa”

Kalle Hirvonen, Alan de Brauw, and Gashaw T. Abate

January 2021

Note: The material contained herein is supplementary to the article named in the title and published in the *American Journal of Agricultural Economics*.

Sampling approach

The sampling frame for the 2019 baseline survey in Addis Ababa was based on a 2017 survey with the same households (Melesse et al. 2019) and followed a multi-stage sampling approach. First, a stratified random sampling method was used to select sub-cities and districts (*woredas*) of Addis Ababa for the survey. To do so, sub-cities were grouped according to their welfare level, after which six sub-cities were randomly drawn from these groups. A similar welfare-based stratification was applied when 20 *woredas* were randomly selected from the selected sub-cities. Second, two urban neighborhoods (*ketenas*) from each selected *woreda* were then randomly selected and from each *ketena*, 25 households were randomly selected for interviewing. In total, 930 households were interviewed in August and September 2019 (Wolle et al. 2020).[1]

The same households were revisited for the January and February 2020 endline survey. This time 895 households were interviewed; 96 percent of the households interviewed during the baseline survey in August and September 2019. Table B1 shows summary statistics for key household characteristics based on the January and February 2020 survey data.[2] Forty-five percent of the households were female-headed, which corresponds to the previous estimates by CSA (2018). The average household in our January and February face-to-face survey sample was 4.5 (median = 4). The average household head was 51 years old and she or he had 6.4 years of education. The average Food Consumption Score (WFP 2008) was 68.2 and the average Household Dietary Diversity Score (Swindale and Bilinsky, 2006) was 9.3 food groups.

[1] A replacement household was randomly drawn if the household interviewed in 2017 was not available in 2019.

[2] Since we label the appendix tables included in the paper with the letter A, for ease of exposition we label these tables starting with B.

Description of the Construction of the Asset index

The February 2020 survey instrument asks for household's ownership of 29 different types of assets:

1. Improved charcoal/wood stove
2. Kerosene (butane gas) stove
3. Electric stove
4. Electric mitad
5. Microwave
6. Refrigerator
7. Dish washing machine
8. Washing machine
9. Modern bed
10. Sofa/couch
11. Wardrobe (cupboard)
12. Modern chair
13. Modern table
14. Land-line telephone
15. Mobile telephone
16. Radio/ tape recorder
17. Television
18. Satellite dish
19. Electric fan
20. Bicycle
21. Motorbike
22. Bajaj
23. Car or truck (vehicle)
24. Computer (laptop or desktop)
25. Electricity (kotari)
26. Livestock
27. Wrist watch
28. Water tanker
29. Water pump

Following Sahn and Stifel (2003), we reduced the asset owned by households into one 'asset index' using principal components analysis (PCA). The 29 asset variables are highly correlated (average correlation coefficient is 0.11) and the principal components analysis attempts to find components that account for most of the variation among these asset types. The end-product is a single variable that we took to represent household asset levels.

We first calculated the eigenvalues for each of the 29 components. The 10 components explain 59 percent of the variation in the data. We then followed the Kaiser-rule that states that only components that obtain an eigenvalue larger than one should be retained. There were 9 such components (Figure B1).

Table B2 provides the principal component loadings based on the first nine components. The Kaiser-Meyer-Olkin (KMO) measure of sampling adequacy is displayed in the last column. We see that many KMO values are close to one, suggesting that the asset categories are indeed measuring a common component. The overall KMO value is 0.82 indicating good sampling adequacy for PCA.

Figure B2 shows the full distribution of the asset index for the 600 households used in the analysis. The asset index ranges between -6.2 and 6.3 with 0.0. Finally, in Figure 2 of the main text we group the households into quintiles based on this asset index. In Figure 4, we rescale the asset index so that it obtains values between 0 and 10.

Comparing characteristics of survey households from the January and February 2020 survey sample that were and were not included in the May 2020 phone survey

Table B3 provides means for selected household characteristics from the January and February 2020 Addis Ababa food consumption survey for the households included in the May 2020 phone survey (N=600) and for the households from the sample for the earlier survey that were not selected to take part in the phone survey. We see that the two sub-samples are generally well balanced. The differences in means are not statistically different from zero, except for the age of the household head, for which the p-value is significant at the ten percent level. The household heads in the

sample included in the phone survey are about two years younger, on average, than households that were not included in the phone survey sample.

Table B1. Basic Household Characteristics, January and February 2020 Survey

	Mean	Median	Standard deviation	Minimum	Maximum
Household size	4.54	4.0	1.9	1	13
Female-headed household	0.45	n/a	n/a	0	1
Head's age in years	51.2	50.0	15.4	11	92
Head's education in years	6.42	7.0	4.6	0	13
Food Consumption Score	68.2	64.0	20.9	8.5	112
Household Dietary Diversity Score	9.27	10.0	1.6	4	12

Note N = 895 households.

Table B2. Sampling Adequacy Based on Kaiser-Meyer-Olkin (KMO) Measure

Asset type	Comp 1	Comp 2	Comp 3	Comp 4	Comp 5	Comp 6	Comp 7	Comp 8	Comp 9	KMO measure
Improved charcoal/wood stove	-0.03	-0.02	-0.07	0.05	0.42	-0.05	-0.20	-0.09	0.60	0.46
Kerosene (butane gas) stove	-0.02	0.10	-0.14	0.30	-0.40	0.05	-0.41	0.27	0.17	0.51
Electric stove	0.25	-0.18	0.11	-0.30	0.08	-0.03	0.17	-0.06	0.01	0.88
Electric mitad	0.30	-0.19	0.09	-0.25	0.02	0.13	0.03	0.03	-0.04	0.84
Microwave	0.12	0.28	-0.11	-0.03	0.17	-0.25	-0.02	-0.03	-0.17	0.81
Refrigerator	0.28	-0.10	-0.02	-0.12	0.01	-0.01	-0.09	0.08	0.03	0.93
Dish washing machine	0.12	0.18	-0.07	0.02	0.17	-0.11	0.02	0.45	0.17	0.86
Washing machine	0.20	0.15	-0.20	-0.16	0.14	-0.27	-0.06	-0.02	0.19	0.87
Modern bed	0.19	-0.09	0.00	0.34	0.31	0.11	0.00	-0.13	-0.07	0.87
Sofa/couch	0.28	-0.14	0.00	0.00	-0.02	0.23	-0.12	-0.04	0.02	0.87
Wardrobe (cupboard)	0.28	-0.10	-0.01	0.06	-0.06	0.08	-0.11	0.02	0.01	0.93
Modern chair	0.14	0.12	-0.31	0.31	0.25	0.18	0.16	0.02	-0.22	0.68
Modern table	0.23	-0.05	-0.21	0.30	0.17	0.32	0.04	-0.01	-0.13	0.79
Land-line telephone	0.17	0.06	-0.07	-0.02	-0.30	0.39	-0.03	0.15	0.00	0.86
Mobile telephone	0.08	-0.05	0.09	0.21	-0.12	-0.30	0.49	0.03	-0.13	0.81
Radio/ tape recorder	0.11	0.07	-0.02	0.08	-0.33	0.02	0.44	0.12	0.34	0.83
Television	0.24	-0.23	0.24	0.30	-0.07	-0.33	-0.13	0.00	-0.02	0.71
Satellite dish	0.25	-0.23	0.24	0.26	-0.08	-0.34	-0.19	0.00	0.01	0.72
Electric fan	0.09	0.34	0.37	0.01	-0.04	0.14	-0.08	-0.17	-0.05	0.71
Bicycle	0.12	0.22	0.04	-0.04	-0.18	0.00	-0.21	-0.26	0.16	0.85
Motorbike	0.05	0.27	0.42	0.05	0.05	0.11	-0.03	-0.20	-0.08	0.69
Bajaj	0.05	0.20	0.30	-0.03	0.16	0.03	0.14	0.51	0.01	0.72
Car or truck (vehicle)	0.17	0.28	-0.14	-0.08	-0.17	-0.13	-0.07	-0.20	-0.26	0.83
Computer (laptop or desktop)	0.22	0.17	-0.13	-0.08	-0.03	-0.10	0.03	-0.20	0.12	0.89
Electricity (kotari)	0.27	-0.16	0.07	-0.33	0.03	0.16	0.04	0.01	0.09	0.84
Livestock	0.02	0.18	0.10	0.24	0.01	0.13	0.31	-0.32	0.38	0.66
Wrist watch	0.20	0.09	-0.13	0.01	-0.21	-0.03	0.16	0.05	0.16	0.90
Water tanker	0.20	0.29	-0.22	-0.10	0.02	-0.21	-0.03	0.05	-0.14	0.85
Water pump	0.06	0.27	0.31	0.03	0.17	0.11	-0.10	0.25	-0.02	0.74
**Overall**										**0.82**

Table B3. Comparing Pre-Pandemic Household Characteristics between Households from the January and February 2020 Survey Sample that Were and Were Not Included in the May 2020 Phone Survey

	Included	Not included		
Number of households:	*600*	*295*		
	**Mean**	**Mean**	**Difference**	**p-value**
Household asset index	3.62	3.62	0.00	0.965
Household size	4.51	4.60	-0.08	0.547
Female-headed household	0.46	0.44	0.02	0.547
Head's age, years	50.4	52.7	-2.2	0.058
Head's education, years	6.57	6.11	0.46	0.153
Food Consumption Score	67.82	68.87	-1.05	0.471
Household Dietary Diversity Score	9.31	9.20	0.12	0.275

Note: Statistical significance tested using a two-sample t-test with standard errors clustered at the enumeration area level.

Table B4. Mean Household Size and Dependency Ratio, by Survey Round

	September 2019	February 2020	August 2020
Household size	5.06	4.52 ***	4.73 ***
Dependency ratio	0.32	0.33	0.31
Number of households	577	577	577

Note: Difference in means tested against the September 2019 value. Statistical significance denoted with * $p < 0.10$, ** $p < 0.05$, *** $p < 0.01$. Dependency ratio is calculated by adding the number of household members who are less than 15 years and the number of household members who are above 65 years and dividing this sum with the total number of household members.

Table B5. Mean Household Diet Diversity Score, by Survey Round and Household Asset Quintile

Survey round	N	Poorest	Poorer	Middle	Richer	Richest	All
September-2019	600	8.4	9.3	9.3	9.4	9.7	9.2
February-2020	600	8.2	9.1	9.5	9.7	10.1	9.3
May-2020	600	7.5	7.9	8.7	9.1	9.4	8.5
June-2020	589	7.6	8.4	8.4	8.9	9.3	8.5
July-2020	584	7.3	8.3	7.9	8.2	8.7	8.1
August-2020	577	8.6	9.5	9.4	9.7	9.9	9.4

Note: Asset quintiles computed from asset index based household asset ownership in February 2020.

Table B6. Change in Mean Weekly per Capita Consumption in Birr between September 2019 and August 2020, by Food Group and Job Loss Status in July

	Job loss	No job loss	Difference in differences
Staples	10.61	8.17	-2.44
Legumes and nuts	-4.46	-2.40	2.06
Vegetables	-13.98	-8.45	5.53
Fruit	3.08	1.26	-1.82
Meat and eggs	17.54	-2.01	-19.55 *
Dairy products	-2.33	-1.21	1.12
All other foods	3.37	0.97	-2.41
**Total**	**13.84**	**-3.67**	**-17.51**

Note: N=577 households in both rounds. Difference in means between the groups tested with a t-test (null-hypothesis: difference in means = 0). Statistical significance denoted with * $p < 0.10$, ** $p < 0.05$, *** $p < 0.01$. Household incurred a job loss if it reported a voluntary or involuntary job loss in the month preceding the July survey (see Figure 3).

Table B7. Change in Mean Daily per Capita Calorie Consumption (in kcal) between September 2019 and August 2020, by Food Group and Job Loss Status in July

	Job loss	No job loss	Difference in differences
Staples	258.9	218.6	-40.3
Legumes and nuts	-37.2	-23.7	13.5
Vegetables	-31.9	-27.0	4.9
Fruit	9.2	4.3	-4.9
Meat and eggs	11.0	-3.6	-14.5 *
Dairy products	2.9	6.5	3.6
All other foods	-51.7	3.2	54.9 **
**Total**	**161.2**	**178.2**	**17.1**

Note: N=577 households in both rounds. Difference in means between the groups tested with a t-test (null-hypothesis: difference in means = 0). Statistical significance denoted with * $p < 0.10$, ** $p < 0.05$, *** $p < 0.01$. Household incurred a job loss if it reported a voluntary or involuntary job loss in the month preceding the July survey (see Figure 3).

Figure B1. Scree plot of eigenvalues after principal components analysis

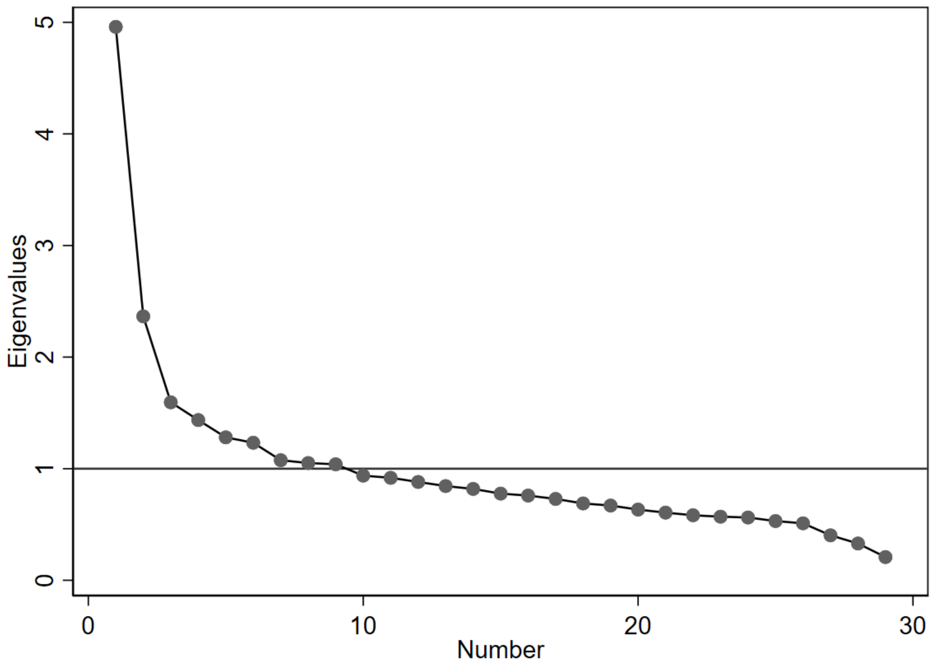

Figure B2. Distribution of the asset index based on Principal Components Analysis (PCA)

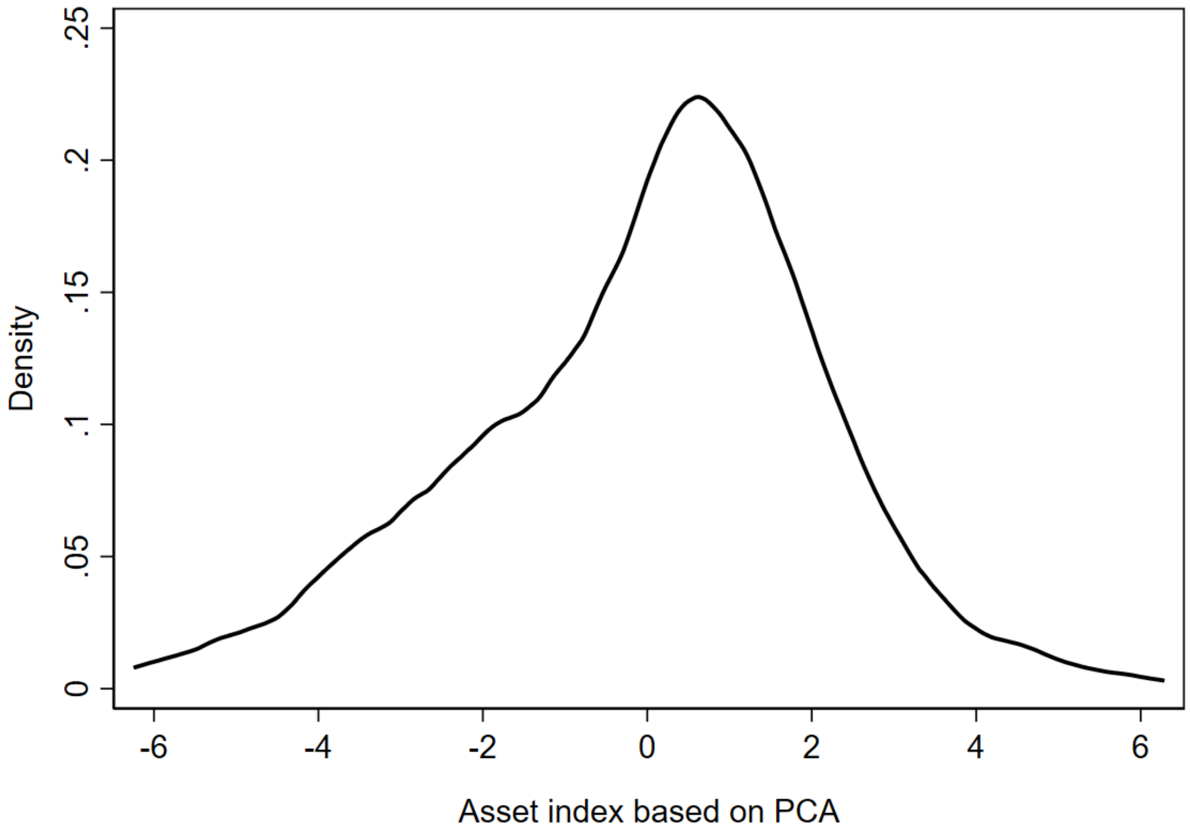

Note: N = 600 households.

Figure B3. Household per capita consumption (in birr) distributions in February-2020 and August-2020

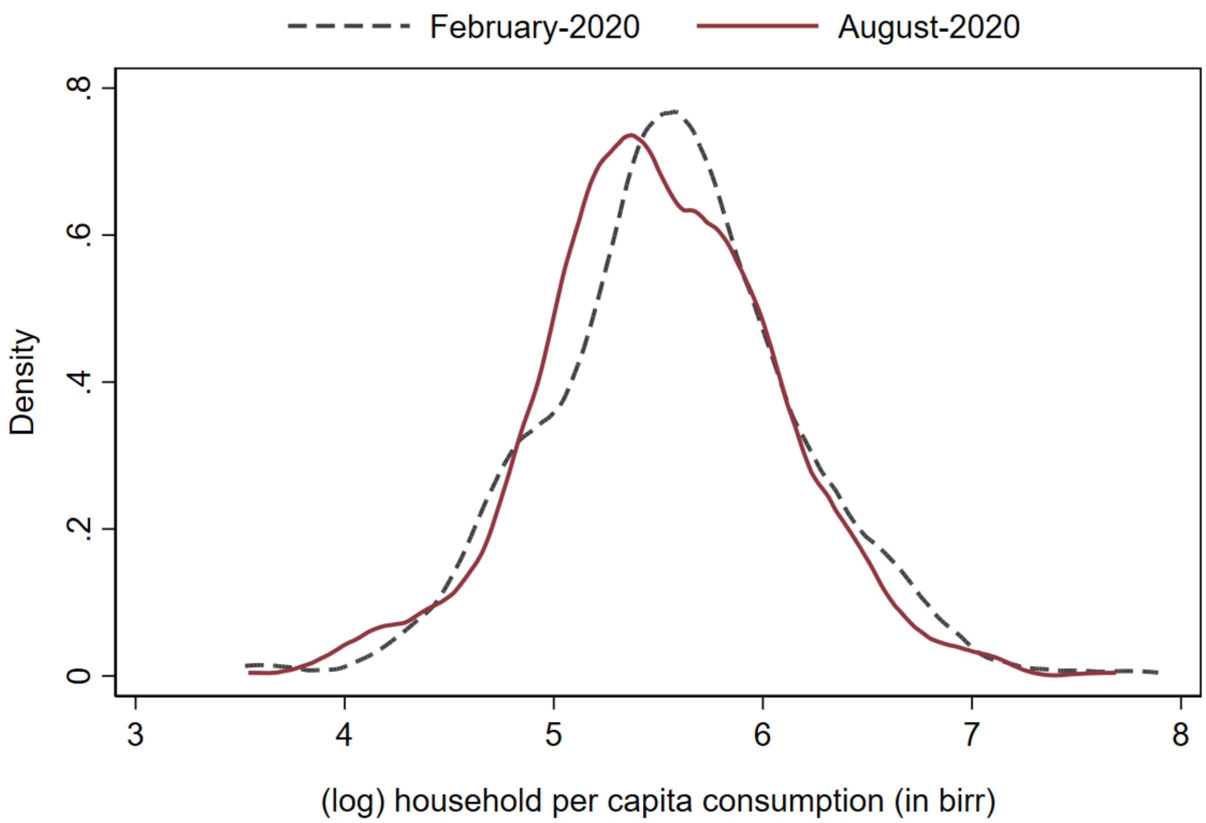

N=577 households in both rounds.

Figure B4. Household per capita consumption (in kcal) distributions in February-2019 and August-2020

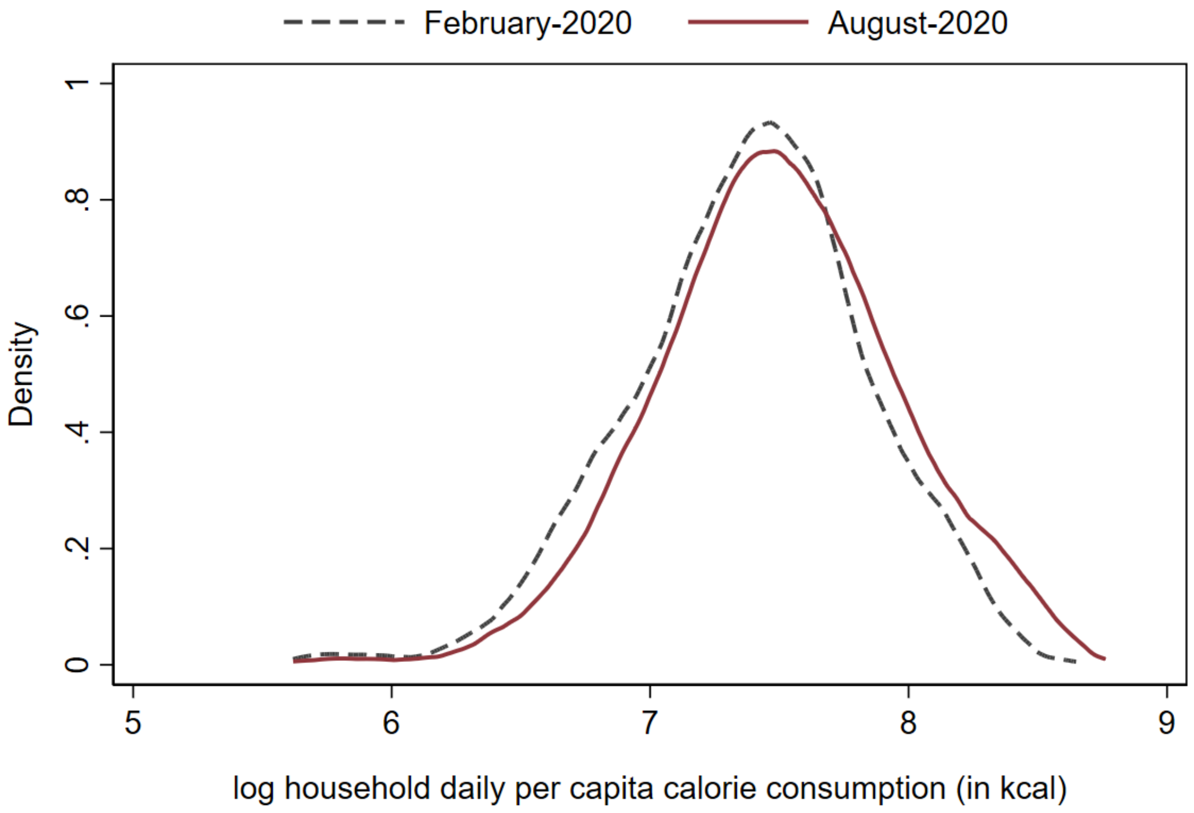

N=577 households in both rounds.
