# Supplementary material for: Food Consumption and Food Security during the COVID‐19 Pandemic in Addis Ababa
Source: Am J Agric Econ. 2021 Feb 23;103(3):772–89. doi: 10.1111/ajae.12206 (PMC8013419; doi:10.1111/ajae.12206)
Supplement: Supplementary file 1 — Appendix S1. Supporting Information. [file AJAE-103-772-s001.zip › AJAE MS#20450-Data and Codes-de Brauw.zip!/replication_files/README.docx]

## Overview

The code in this replication package constructs the analysis file for **"Food Consumption and Food Security during the COVID-19 Pandemic in Addis Ababa**" (2021) by Kalle Hirvonen, Alan de Brauw, and Gashaw T. Abate using Stata.

Stata do file called 'Hirvonen_deBrauw_Abate_2021_AJAE_tables_figures.do' provides the code by calling 'dta' folder that contains 8 Stata dta files. The replicator should expect the code to run for less than 2 minutes.

## Data Availability and Provenance Statements

The data used in the manuscript are household survey data collected by the authors in 2020 from Addis Ababa Ethiopia, except for the Consumer Price Data used in Appendix G that was compiled by the Central Statistical Agency of Ethiopia. All primary household survey data used in this paper will be de-identified and made publicly available 2 years after the completion of the project (i.e., in 2023). The data provided here permits a narrow replication (i.e., “push-button” replication of the results).

### Statement about Rights

- We certify that the authors of the manuscript have legitimate access to and permission to use the data used in this manuscript.

### Details on each Data Source

The "dta" folder contains 8 Stata dta files:

- "shocks.dta":
  - This dta file is based on data collected in May, June and July phone surveys.
  - It provides information about the income shocks and job losses experienced by the households in month preceding the interview.
  - The data are at the household, survey round level with unique identifiers: *HH_ID round.*
- "diets.dta"
  - This dta file is based on data collected in all 6 survey rounds.
  - It provides the Household Diet Diversity Score (HDDS).
  - The data are at the household, survey round level with unique identifiers: *HH_ID round.*
- "food_consumption_levels.dta"
  - This dta file is based on data collected in September-2019, February-2020 and August-2020 survey rounds.
  - It provides the data on household food consumption in the 7-day period prior to the interview, both in Ethiopian birr and kcal terms.
  - The data are at the household, survey round level with unique identifiers: *HH_ID round.*
- "food_consumption_changes.dta"
  - This dta file is based on data collected in September-2019 and August-2020 survey rounds.
  - It provides the data on changes in household food consumption between the two survey rounds, both in Ethiopian birr and kcal terms.
  - The data are at the household level with unique identifiers: *HH_ID.*
- "Figure_4_data.dta"
  - This dta file is based on data collected in all 6 survey rounds.
  - It contains the means and confidence intervals of HDDS for all 6 rounds.
  - The data are at the survey round level with unique identifier: *id.*
- "assets.dta"
  - This dta file is based on data collected in February 2020 round.
  - It contains information about the assets owned by the households.
  - The data are at the household level with unique identifier: *HH_ID.*
- "february2020_sample.dta"
  - This dta file is based on data collected in February 2020 round.
  - It contains information about the basic characteristics of the households.
  - The data are at the household level with unique identifier: *HH_ID.*
- "prices.dta"
  - This dta file is based on Consumer Price Index (CPI) for Addis Ababa, compiled by Central Statistical Agency of Ethiopia.
  - It contains the price index based on CPI and consumption weights from our household survey administered in September-2019.
  - The data are at food group month round level with unique identifiers: *FG year month.*

Each dta file is self-describing, containing both variable and value labels.

### Software Requirements

Stata (code was last run with version 16.1)

### Memory and Runtime Requirements

The script runs with a standard (2021) desktop or laptop machine. Approximate time needed to reproduce the analyses is less than 5 minutes.

## Description of programs/code

Stata do file called "Hirvonen_deBrauw_Abate_2021_AJAE_tables_figures.do" runs all analyses.

## Instructions to Replicators

- Use 'cd' command to set the working directory (i.e., the folder in which the replication package is stored in).
- The do file will store the log file into folder called 'log_file'
- The do file will call dta files from the folder called 'dta' and reproduce the tables and figures.
- Results are stored to folder called 'results', all tables (except Table 2) are displayed on the screen.
