# Supplementary material for: Food Consumption and Food Security during the COVID‐19 Pandemic in Addis Ababa
Source: Am J Agric Econ. 2021 Feb 23;103(3):772–89. doi: 10.1111/ajae.12206 (PMC8013419; doi:10.1111/ajae.12206)
Supplement: Supplementary file 1 — Appendix S1. Supporting Information. [file AJAE-103-772-s001.zip › AJAE MS#20450-Data and Codes-de Brauw.zip!/replication_files/results/Table_2.rtf]

Table 2. Association between self-reported income shock and Household Diet Diversity Score, household fixed effects regression
	(1)	(2)	
	HDDS	HDDS	
Income shock	-0.053	-0.031	
	(0.095)	(0.091)	
Observations	1773	1773	
Standard errors in parentheses
^*^ p < 0.10, ^**^ p < 0.05, ^***^ p < 0.01
